# Heat the Clock: Entrainment and Compensation in *Arabidopsis* Circadian Rhythms

**DOI:** 10.5334/jcr.179

**Published:** 2019-05-14

**Authors:** Paula A. Avello, Seth J. Davis, James Ronald, Jonathan W. Pitchford

**Affiliations:** 1 Department of Mathematics, University of York, UK; 2 Department of Biology, University of York, UK

**Keywords:** circadian clock, mathematical modelling, temperature entrainment, temperature compensation, diurnal temperature range

## Abstract

The circadian clock is a biological mechanism that permits some organisms to anticipate daily environmental variations. This clock generates biological rhythms, which can be reset by environmental cues such as cycles of light or temperature, a process known as entrainment. After entrainment, circadian rhythms typically persist with approximately 24 hours periodicity in free-running conditions, *i.e.* in the absence of environmental cues. Experimental evidence also shows that a free-running period close to 24 hours is maintained across a range of temperatures, a process known as temperature compensation. In the plant *Arabidopsis*, the effect of light on the circadian system has been widely studied and successfully modelled mathematically. However, the role of temperature in periodicity, and the relationship between entrainment and compensation, are not fully understood. Here we adapt recent models to incorporate temperature dependence by applying Arrhenius equations to the parameters of the models that characterize transcription, translation, and degradation rates. We show that the resulting models can exhibit thermal entrainment and temperature compensation, but that these phenomena emerge from physiologically different sets of processes. Further simulations combining thermal and photic forcing in more realistic scenarios clearly distinguish between the processes of entrainment and compensation, and reveal temperature compensation as an emergent property which can arise as a result of multiple temperature-dependent interactions. Our results consistently point to the thermal sensitivity of degradation rates as driving compensation and entrainment across a range of conditions.

## Introduction

The circadian clock is an interconnected network of biological processes needed for some organisms to anticipate daily environmental variations. The synchronization of the clock with the night/day cycle grants advantages such as growth and development in plants [1,2,3,4,5,6], energy balance in mammals [7], conidium development in Neurospora [8], sleep modulation in Drosophila [9] and starvation response in Cyanobacteria [10]. This biological system generates rhythmic gene expression, and mathematical models have helped to uncover the crucial molecular mechanisms of diverse living organisms [11,12,13,14,15,16,17,18,19,20,21]. For an overview of insights into the complexity of circadian systems using mathematical and biological techniques, see [22,23,24,25,26].

In plants, mathematical models based on ordinary differential equations (ODEs) were built in order to characterize the first feedback loop identified by experimental observations. Subsequently, mathematical models have continued describing the key mechanisms driving the plant oscillator [11,12,13,27,28,29,30,31,32,33,34]. These models have motivated hypotheses which, in parallel with experimental validation, have helped to establish not just the components of the network, but also to elucidate their role [24,35].

Light and temperature are important stimuli to regulate circadian rhythms [36,37]. However, most modelling efforts have only focused on incorporating the effect of light in the plant circadian system. The influence of temperature is less clear, and is less well studied. A better understanding of temperature dependence in the circadian clock, and its relation to light, is needed for several reasons. While experiments necessarily concentrate on controlled and idealised scenarios, the real physiological challenges faced by plants are more varied. In the simplest case, global climate warming will require plants to maintain a 24 hour rhythm at an increased average temperature. Alternatively, a local environmental warming might require a plant to disperse toward a higher latitude to maintain an optimal temperature range. Such a latitudinal change will necessarily involve a change in the associated light-dark cycle, with higher latitudes being subject to (in summer) longer days and shorter nights. Similarly, for crop plants, it may be the case that potentially productive cultivars have evolved at different latitudes or temperatures; an understanding of how a plant’s clock will function when translocated to a new environment could be crucial in assessing its suitability [38,39].

An important property of many circadian clocks is that the free-running period, i.e. the frequency of the oscillator in the absence of changing external stimuli, varies minimally over a range of temperatures, a phenomenon known as temperature compensation. A small number of studies consider the role of temperature in the Arabidopsis circadian clock, seeking to combine experimental results with numerical simulations. [40] simulated the role of *GIGANTEA (GI)* in temperature compensation using the model of [33]. By modifying the transcription rates of the genes *CIRCADIAN CLOCK ASSOCIATED (CCA1) and LATE ELONGATED HYPOCOTYL (LHY)* along with the hypothetical gene Y, they were able to fit the experimental data and to propose that *GI* is a component of Y. Also, [41] used [33] model to test the contribution of *FLOWERING LOCUS C (FLC)* on compensation observed in experiments. They could fit experimental observations by increasing the maximum transcription rate of the hypothetical gene X in the model. This simulated the *LUX ARRHYTHMO (LUX)* gene effects on the clock, which is known to play a role in temperature compensation [42]. [43] built a temperature compensated model by incorporating temperature dependence into the [28] model. The authors thereby explained temperature compensation as a consequence of the architecture of the clock network, where rates of transcription, translation and, mRNA and protein degradation were hypothesised to change with temperature.

Explanations of temperature compensation are centred around two hypotheses [43,44]. The first proposes that the structure of the clock gene network, together with simple temperature dependence of its control coefficients, have evolved in order to produce an overall balance. The alternative hypothesis is that separate specific molecular mechanisms have evolved in order to ensure compensation, as argued in the case of the Neurospora circadian clock [45]. It is notable, however, that [45] hypothesised temperature dependence of two key translation rates using idealised hyperbolic tangent functions. This is in contrast to the Arrhenius formulation used in [43]. The latter approach, which allows for a simple parameterisation and which can be derived from the laws of statistical mechanics, forms the basis of this investigation.

The ability to maintain a 24 hour free-running period across a range of temperatures may not be a property subject to evolutionary selection; of more practical ecological relevance is an ability to maintain a circadian rhythm under a range of varying, and perhaps unpredictable, light and temperature stimuli. In this sense, although the mechanisms driving them may be related, the ability of a clock to be entrained must be separated from the phenomenon of temperature compensation. Mathematical models allow precisely this separation to be achieved.

Here we aimed to gain insight into clock mechanisms involved in adaptation to changing environmental conditions. To this end, we modified a recent model [11] by hypothesising temperature-induced changes in reaction rates, distinguishing the roles of transcription, translation and degradation. We conducted a simulation study across a wide range of light/dark and warm/cold regimes, and we explored the ability of our model to be thermally entrained and to show temperature compensation. Our results generally supported the holistic network-driven hypothesis for compensation, but they emphasised the importance of the thermal dependence of degradation on the clock’s function. These conclusions were generally supported when the results were challenged by allowing greater uncertainty in the thermally sensitive parameters, and when results were compared to those from an earlier, more complex, mathematical model [27].

## Methods

### The model

**Figure 1:**
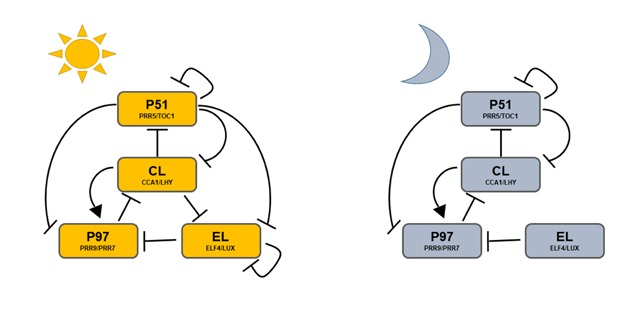
**Schematic diagram illustrating main features of the [11] model in light and dark phases.** In the model, similar genes were merged into the single variables CL, P97, P51, and EL, which represent the pair of genes *CCA1/LHY, PRR9/PRR7, PRR5/TOC1*, and *ELF4/LUX*, respectively. Edges represent transcription factors affecting the transcription rates of a gene in the network.

The mathematical model presented here is based on [11]. The model consists of nine coupled ordinary differential equations (ODEs) describing changing protein and mRNA levels, as detailed in Supplementary Material. The model is considerably simpler than earlier models [12,13,27,28], but is known to replicate many key features of the plant circadian system. The model describes the dynamics of eight genes grouped into pairs, plus a dark-accumulating protein P, which represents the proteins PHYTOCHROME INTERACTING FACTOR 3 (PIF3) and PHYTOCHROME INTERACTING FACTOR 3-LIKE 1 (PIL1). The pairs of clock genes are: *CCA1/LHY*, the *PSEUDO-RESPONSE REGULATOR 7* and *9* genes (*PRR9/PRR7*), the evening genes *EARLY FLOWERING 4* and *LUX (ELF4/LUX)*, and *PSEUDO-RESPONSE REGULATOR 5* with *TIMING OF CAB EXPRESSION 1 (PRR5/TOC1)*. Figure 1, adapted from Figure 1 of [11], depicts the structure of the network and shows how its components interact via positive and negative feedbacks.

The ODE system proposed by [11] yields a total of 36 parameters (Table 1 in Supplementary Material), which represent transcription, degradation and translation rates associated with mRNA and protein levels of the pairs of genes included in the model, as well as the effect of light. Light regulates all model components at mRNA or protein level and its effect is parametrized in a binary manner: forcing variables L and D take values L = 1 and D = 0 in light phases, and L = 0 and D = 1 in darkness.

As explained in [11], the parameter values of the model are based on a fit to qualitative dynamics, based on matching amplitude, phase and period with experimental observation. Details of the model and its parameter values are in the Supplementary Material. All simulations and analysis were performed in MATLAB. The integration of the system of ODEs was performed using the ODE23s solver, and numerical accuracy was verified by comparing to the outputs of alternative solvers such as ODE45.

### Including temperature dependence

The original model of [11] includes light as the only external stimulus, thereby limiting any entrainment response to be purely driven by light. In order to incorporate temperature entrainment and to assess compensation, we introduce temperature dependence by assuming that each rate can be described by an Arrhenius equation [46,47,48]. This equation describes a temperature dependent reaction rate via the concept of an activation energy, defined as the minimum amount of energy needed for a reaction to occur (Figure 1 in Supplementary Material). A closely related concept is the *Q*_10_ coefficient. *Q*_10_ is defined as the ratio of the reaction rates measured at two temperatures differing by 10°C [49].

Explicitly we assume that, for a given rate *i*,

1 {k_i} = {A_{i}}\,{\rm{exp}} \left({\frac{{ - {E_i}}}{{RT}}} \right),

where the parameters *A_i_* and *E_i_* are physical constants, respectively a rate constant and an activation energy for that particular reaction, and *R* is the universal gas constant (8.3145 × 10^–3^ kJ mol^–1^ K^–1^) and *T* is temperature.

This formulation appears to introduce a further two unknown parameters for each temperature dependent reaction. However, by requiring that the model parameters match those fitted by [11] at their reference temperature of 21°C, and by defining a realistic *Q*_10_ value for each reaction, this apparent uncertainty is ameliorated. Explicitly, an expression for activation energy can be determined from the *Q*_10_ temperature coefficient which is defined by,

2 {Q_{10}} = {\left({\frac{{k_i^{{T_2}}}}{{k_i^{{T_1}}}}} \right)^{\frac{{10^\circ }}{{{T_2} - {T_1}}}}},\,\,\,{T_2} > {T_1}.

Combining equations 1 and 2 gives

3 {E_i} = \frac{{R \times \,{\rm{log}} \left({\frac{{k_i^{{T_2}}}}{{k_i^{{T_1}}}}} \right)}}{{\frac{1}{{{T_1}}} - \frac{1}{{{T_2}}}}},

and

4 {A_{i}} = {k_i} \exp \left({\frac{{{E_i}}}{{RT}}} \right).

This equation immediately allows an indicative value of *E_i_* to be calculated: assuming a reference temperature of *T*_1_ = 21°C, and a *Q*_10_ of 2, substituting into equation (3) gives an approximate value of *E_i_* = 50 kJ mol^–1^. This value of the activation energy is used as a starting point in the following numerical study. The role of the incertainty in the values of *E_i_* for each reaction *i* can be investigated via Monte Carlo simulations spanning a range of plausible values, see Supplementary Material.

Alternatively, *Q*_10_ is defined in terms of the period length P [50],

5 {Q_{10}} = {\left({\frac{{{P_{{T_1}}}}}{{{P_{{T_2}}}}}} \right)^{\,\frac{{10^\circ }}{{{T_2} - {T_1}}}}},{\rm{}}{T_2} > {T_1}.

Therefore, overcompensation is reflected for *Q*_10_ < 1, (i.e. period length increases as temperature rises). In contrast, for undercompensation cases, a *Q*_10_ > 1 is observed (i.e. period length shortens with increasing temperature).

### Combining light and temperature variation

The preceding mathematics allows simultaneous changes in both temperature and light to be simulated in the model, and their roles to be quantified. The structure of the numerical investigation is as follows: Initially, the behaviour of the temperature dependent model is studied in two general contexts: temperature entrainment (i.e. can meaningful 24 h cycles be induced by realistic daily variation in temperature) and temperature compensation (i.e. how sensitive to temperature is the free-running period of the clock). Thereafter, simultaneous changes in light and temperature are imposed, to investigate under what environmental conditions a circadian rhythm is predicted to remain viable.

## Results

### Temperature entrainment

Simulations were carried out to replicate standard experimental conditions [29,51]. These were the model was entrained for 7 days under 12 h warm/12 h cold temperature cycles (a diurnal temperature range of 4°C was simulated (4°C difference between warm and cold)) under constant light before release into free-running conditions (constant light and warm temperature).

**Figure 2:**
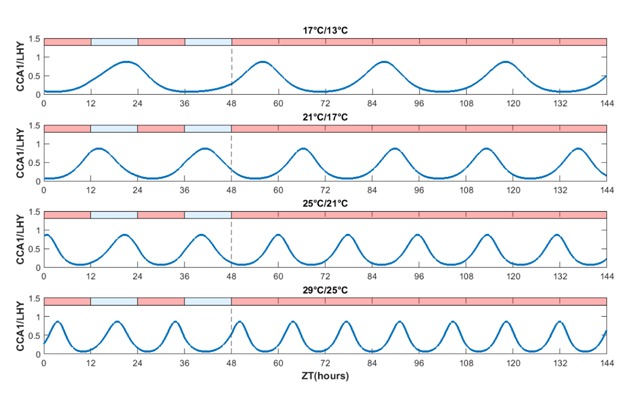
**Incorporating the Arrhenius law allows thermal entrainment, but only within a limited temperature range.** A 24 hour 21°C/17°C thermal cycle induces a functional clock, as shown by the rhythmic expression of *CCA1/LHY* mRNA. However, a 17°C/13°C thermal cycle induces a markedly increased period while warmer temperatures cause faster oscillations and ultradian rhythms.

Figure 2 shows the predicted gene expression (mRNA levels) of *CCA1/LHY* in the last two thermal cycles, and for the four days following the release of the clock into free-running conditions. The key features of the output can be divided into two regions, referring to behaviour under thermal entrainment (up to ZT48 in the figure), and then to subsequent free-running behaviour (after ZT48).

**Figure 3:**
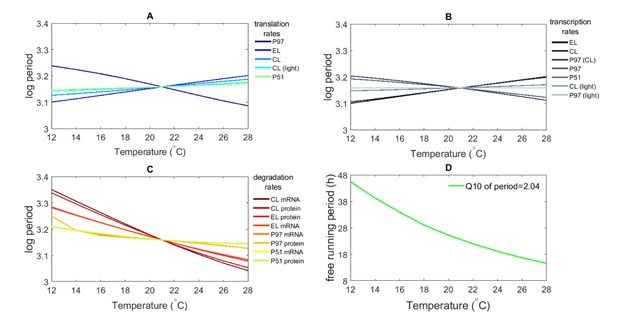
**The model is not temperature compensated, and this failure to compensate is driven by degradation rates.** Experimental protocols of [40] and [53] were simulated. A–C show the log period across a range of temperature, where only one parameter is subject to temperature variation for each output. Results are grouped to show thermal dependence of **(A)** translation, **(B)** transcription, and **(C)** mRNA and protein degradation rates. Labels are in order of the size of effect and they show how the parameter’s effects are induced (in brackets). The resultant free-running period when all rates vary with temperature is shown in **(D)**. Note that thermal dependence in translation or transcription may either increase or decrease period, whereas changing degradation rates causes a consistent decrease.

The second row of outputs, depicting a moderate thermal forcing of 12 h at 21°C followed by 12 h at 17°C, shows that the clock can be thermally entrained; the clock oscillates approximately once per thermo-cycle. However, thermal entrainment to a 24 h period was not observed in cooler scenarios (first row: at 17°C/13°C the observed period is 34.3 h), and nor was it observed under warmer scenarios sharing the same 4°C variation (third row: at 25°C/21°C the clock’s period is significantly shorter than 24 h; fourth row: at 29°C/25°C the thermally entrained rhythm is approximately 15 h). This behaviour is not unique for *CCA1/LHY*; similar results were observed for *ELF4/LUX, PRR9/PRR7*, and *PRR5/TOC1* (Figures 2, 3, and 4 in Supplementary Material).

A similar pattern emerges when examining the behaviour of the entrained (or otherwise) clock under subsequent free-running conditions. After ZT48, the clock held at 21°C displays an approximately 24 h period (second row), but systems held at cooler and warmer temperatures display large variation from this circadian behaviour, with periods in excess of 30 h at 17°C and shorter than 14 h at 29°C.

To further test the applicability of the model, its behaviour under severe temperature stress conditions in constant light was also analyzed. Consistent with results in Figure 2, at 24 hour 5°C/1°C thermal cycle (freezing stress) the clock showed a pronounced increased period, while at 39°C/35°C thermal cycle (heat stress) the clock displayed substantially higher frequency oscillations (Figure 5 in Supplementary Material).

The consistent story which emerges is that thermal entrainment may be possible in the absence of light/dark forcing, but only within a limited temperature range (where the free-running period is approximately 24 h in any case). In other words, it is the interaction between light and temperature, rather than temperature in isolation, which is important in understanding the robustness of circadian rhythms in varying environments. To test that this general conclusion is not sensitive to the exact choice of the activation energies *E_i_* we also carried out simulations where each activation energy was chosen at random, independently, from a U [40,60] distribution (see Figure 6 in Supplementary Material).

### Temperature compensation

Temperature compensation has been explained as emergent property, happening via a balance of network reactions each of which is not necessarily compensated [52]. That the [11] model can in principle display temperature compensation, but that this is not the case in general, is illustrated in Figure 3. Figure 3D shows that when each constituent reaction in the network is allowed to vary with temperature with *Q*_10_ = 2, the resulting clock has free-running period which declines markedly with temperature (undercompensation). The resultant *Q*_10_ of period value is around 2.

The reasons for this lack of overall compensation can be elucidated by running a modified model allowing temperature dependence (with *Q*_10_ = 2) in only one rate in any given simulation [48]. Figure 3A–C summarises this, where the resultant free-running periods from allowing thermal dependence of each of the 20 parameters in isolation is plotted. The outputs are grouped into thermal dependence of (A) translation rates, (B) transcription rates, and (C) mRNA and protein degradation rates. Figure 3A and B show that translation and transcription rates can have both positive and negative effects on period. For example, in Figure 3A an increase in the translation rate of PRR9/PRR7 shortens the period with increasing temperature, whereas the period increases with temperature for all other represented genes. This indicates that any overall temperature compensation model must emerge from a balance between these processes. Indeed, if the model is implemented with thermal variation in all translation rates, or in all transcription rates, the emergent clock is thermally compensated with a *Q*_10_ of period approximately equal to 0.97 in both cases.

Figure 3C shows that, in contrast, thermal dependence of degradation rates results in consistent reductions of period with increasing temperature, and that the influence of these degradation rates exceeds that of the translation and transcription rates. In other words, the overall failure of compensation in Figure 3D can be attributed to the thermal dependence of degradation rates.

### Simultaneous effects of light and temperature

We then simulated the clock in more realistic environmental conditions by simultaneously changing light and temperature. Explicitly, simulations were carried out using a 24 h cycle length, with this 24 h divided into (light and warm) and (cold and dark) regimes of variable durations, where the cold temperature is (respectively) 4°C, 8°C and 12°C lower than the warm temperature. As in Figure 2, all rates were allowed to vary with temperature using an assumption that *E_i_* = 50 kJ mol^–1^ for each reaction.

**Figure 4:**
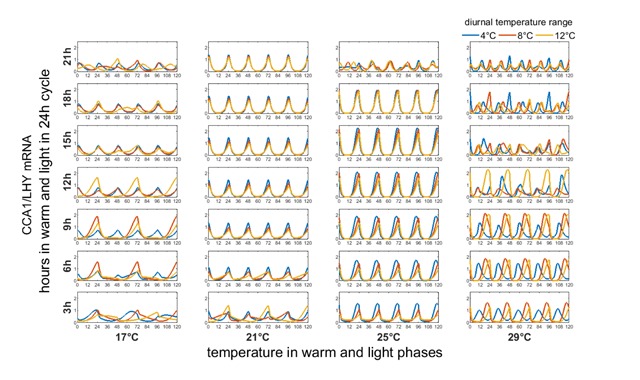
**The combined effect of photic and temperature forcing can induce entrainment across a wide range of light/dark durations, but only within narrow temperature limits.** At 21°C the clock is correctly entrained even for large diurnal temperature ranges and uneven light-dark cycles (central columns). At lower (first column) or higher (final column) temperatures, non-circadian rhythms may emerge. Further, there is a general increase in amplitude as temperature increases.

Figure 4 summarises the results. The central row of the second column depicts a circadian clock under “standard” conditions with 12 h in light at 21°C followed by 12 h dark at cold temperatures cycles, the system is entrained to a predictable 24 h rhythm. This rhythm persists, with increased amplitude, at 25°C, and also at 17°C provided the temperature range does not exceed 8°C.

However, at 29°C the circadian functionality of the clock is lost. Interestingly, when the temperature variation is at its largest (12°C) this has the effect of preventing entrainment at low temperatures (where a 48 h period is induced), but conversely of restoring circadian rhythmicity at 29°C.

In contrast, the relative consistency of outputs in the second column indicates that, even at extreme durations ranging from 6 h to 21 h light, the clock can be successfully thermally and photically entrained at this temperature. The circadian rhythm is disrupted only at the shortest light duration of 3 h. The system can respond to wide changes in light/dark cycles so long as the temperature remains close to 21°C.

The clock was again relatively entrainable at a base temperature of 25°C, but with diminishing amplitude (and eventual failure of the circadian rhythm) at the extremes of simulated light duration (3 h or 21 h). However, at the warmest (29°C, final column) temperatures the circadian clock behaved erratically, with a general decline in amplitude and increase in period as temperature increases. There are signs of erratic ultradian rhythms when the light duration exceeds 12 h, but also of circadian rhythms which display a large phase shift for the shorter light durations.

At cooler temperatures (17°C, first column), the amplitude of oscillations decreased. In short days (3 h, 6 h and 9 h of warm-light) the clock showed long period oscillations, with a transition to circadian dynamics with low amplitude as light duration increases.

## Discussion

Using numerical investigations, we studied how the [11] model clock responds to the combined effects of light and temperature. The results demonstrate that dynamics in both of these environmental processes are instrumental in resultant clock dynamics, and that any theoretical or empirical assessment of the robustness of the clock to these changes needs to be viewed in an ecological context. Moreover, the results clearly distinguish between the processes of entrainment and compensation, and they point to the key elements in the circadian clock which drive both phenomena.

One broad conclusion is that temperature compensation cannot be explained as the result of a balance between a set of temperature dependent reactions within the clock, and that the reason for this is closely linked to temperature dependence of mRNA and protein degradation rates. If degradation rates were relatively insensitive to temperature, then the interaction between temperature dependent rates of translation and/or transcription would be sufficient to explain temperature compensation, supporting the network hypothesis of [43]. However, because there is substantial evidence that degradation rates are temperature sensitive [54], temperature compensation must be achieved by more complex processes (see, for example, [55]) which are beyond the scope of this model.

For the clock to be functional it needs to interact with both light and temperature. From our numerical results, we hypothesise that in nature, the function of the plant’s circadian clock might be adversely affected by long periods of exposure to warm and light conditions. Evidence that temperature compensation in the *Arabidopsis* clock results from the interaction of light and temperature have been published [43]. Moreover, in our approach *PRR9/PRR7* is essential for temperature compensation, which agrees with experimental results [53]. Notably, our approach allows us to present a theoretical climatic tolerance range resulting from entraining the clock for more realistic environmental cues involving light and temperature cycles simultaneously.

To further test whether our outputs from simultaneous photic and thermal cycles were consistent with experimental results, we simulated the clock for a total of 15 days under both 24 h light/warm and dark/cold cycles, and compared these to dark/warm and light/cold cycles, following the experimental ideas of [56] and [57] (Figure 13 in Supplementary Material). Although there are some differences in quantitative details, our model predictions generally matched those results. A circadian rhythm was maintained, and a higher peak expression was observed when the clock was entrained under 25°C in light and 15°C in dark conditions, compared to 15°C in light and 25°C in dark. Furthermore, in 25°C light and 15°C dark the mRNA levels accumulated more rapidly in dark phases, and this increase was slightly advanced, compared with 15°C in light and 25°C in dark entrainment cycles.

The model on which this study is based, while being based on established biological and physical mechanisms, necessarily relies on several assumptions. Perhaps the most restrictive of these are firstly, that each individual component reaction is subject to thermal variation following an identical *Q*_10_ of 2, and secondly, that the underlying model of [11] may be an oversimplification of the true biological system.

The first of these concerns can be addressed via Monte Carlo simulation. Explicitly, in Figures 2, 3, 4 which are calculated under the *Q*_10_ = 2 assumption, each of the activation energies *E_i_* in equation 1 is assumed to take a value of 50 kJ mol^–1^. While this is reasonable as a first assumption, it is likely to be an oversimplification; for example there is evidence of *Q*_10_ coefficients for degradation rates taking values around 3 [54]. This assumption can be relaxed by choosing each *E_i_* independently and at random, for example from a uniform distribution between 40 kJ mol^–1^ and 60 kJ mol^–1^. Figure 6 in Supplementary Material summarises example trajectories for 20 such randomisations from a total of 200 realizations. The *Q*_10_ of rate and *Q*_10_ of period distributions of those 200 random parametrizations are provided in Figure 11 in Supplementary Material; it is clear that, although there are differences in detail between the outputs arising from random parameter sets, the overall qualitative story is unchanged. Naturally, simulating greater variation in activation energies induces a greater range in modelled outputs. While it is impractical to demand empirical work to estimate Arrhenius parameters governing each interaction, theoretical results are useful in emphasising which elements of the circadian clock might be most relevant in driving the overall dynamics. In this case, degradation rates play a surprisingly consistent role in the temperature dependence of the free-running period (Figure 3), and these might form the basis for further useful experimental scrutiny.

The simple model studied here is based on that of [11], and it is known that this relatively simple model is in principle susceptible to non-trivial resonance and chaotic regimes in its response to regular parametric forcing [58]; these may influence the erratic outcomes observed in, for examples, the extreme temperatures in Figure 4. Therefore, to investigate whether the choice of model is critical to the conclusions, we implemented the same theoretical approach to include temperature in the more complex model of [27] (Figures 7, 8, and 9 in Supplementary Material). Again, although differing in some details, very similar results emerge. As with the [11] model, mRNA levels decrease as temperature decreases. Importantly, degradation rates are also key in undercompensation. This support the importance of interactive work needed between modelling and experiments for future investigation. Efforts should be focused on buillding a model that is able to show temperature compensation by including the recent experimental findings, for example, the placement of HSP90 within the clock for temperature behaviour [59] along with ambient temperature effects on degradation rates [54].

Experimental observations for entrainment by temperature cycles have shown that temperature is an important zeitgeber in the plant circadian clock. Even a small change in temperature of 4°C is able to reset the clock [60]. Our results are consistent with this. The simple model presented here has the ability to reproduce sustained oscillations entrained by a relatively small temperature range. Temperature has an important speeding up effect in the entrainment process, and this effect in periodicity is stronger compared to light entrainment [61]. Our clock model mirrors these findings. The free-running period of [11] model (i.e. after photic entrainment) is 23.6 h in constant light [58]. Our model predicts a free-running period of 21.88 h after thermal entrainment when a 24 hour 22°C/16°C thermal cycle is simulated, as observed in [61]. Moreover, results in Figure 2 showed that the period observed under entrained conditions is reduced by approximately 10% (on average across the temperatures analysed) when the clock is released into free-running conditions. In contrast, for photic entrainment, the period is reduced by only 2%. Additionally, *PRR9* and *PRR7* clock components have been revealed as necessary for the plant response to thermal cycles [62]. We simulated the clock for the *prr9prr7* double mutant by dividing the relevant transcription rate by a factor of 10 [11], and similar results to [62] were observed (Figure 10 in Supplementary Material). *CCA1/LHY* expression clearly oscillated in *prr9prr7* double mutant, and showed longer period length compared to wild type. A phase delay was also observed in simulations, consistent with [62].

The principal conclusion of this study is to distinguish between the phenomena of entrainment and compensation; robust temperature compensation depends on the interrelationship of a network of temperature-dependent processes and is particularly sensitive to details of temperature-dependent degradation, whereas entrainment to temperature alone operates within a more confined parameter space. In more realistic conditions where both light and temperature fluctuate, the circadian clock is predicted to show a robustness which would be hidden if each varying factor were to be considered alone.

## Additional Files

The additional files for this article can be found as follows:

10.5334/jcr.179.s1 Figure 1. The larger activation energy, the higher the dependence of the reaction rate on the temperature.

10.5334/jcr.179.s2 Figure 2. Incorporating the Arrhenius law allows thermal entrainment, but only within a limited temperature range.

10.5334/jcr.179.s3 Figure 3. Incorporating the Arrhenius law allows thermal entrainment, but only within a limited temperature range.

10.5334/jcr.179.s4 Figure 4. Incorporating the Arrhenius law allows thermal entrainment, but only within a limited temperature range.

10.5334/jcr.179.s5 Figure 5. Temperature stress under constant light conditions can cause a defective clock.

10.5334/jcr.179.s6 Figure 6. Thermal entrainment is observed in the model across a range of parameter values describing temperature dependence.

10.5334/jcr.179.s7 Figure 7. Incorporating temperature dependence in a more complex model [27] supports the conclusions obtained using the [11] model.

10.5334/jcr.179.s8 Figure 8. The [27] model is not temperature compensated, and this failure to compensate is driven by degradation rates.

10.5334/jcr.179.s9 Figure 9. The combined effect of photic and temperature forcing can induce entrainment across a wide range of light/dark durations, but only within narrow temperature limits.

10.5334/jcr.179.s10 Figure 10. *CCA1/LHY* expression in *prr9prr7* oscillates in response to temperature cycles with a phase shift compared to wild type.

10.5334/jcr.179.s11 Figure 11. A *Q*_10_ of period of around 2 is obtained when random activation energy values between 40 kJ mol^–1^ and 60 kJ mol^–1^ are used.

10.5334/jcr.179.s12 Figure 12. *CCA1/LHY* expression for a diurnal temperature range of 10°C.

10.5334/jcr.179.s13 Figure 13. Simulated *CCA1/LHY* expression qualitatively mirrors experimental data contrasting warm/light and cold/dark with cold/light and warm/dark cycles.

10.5334/jcr.179.s14 The Model. The model is summarised by the following system of ordinary differential equations.

10.5334/jcr.179.s15 Table 1. Parameter values.
