# Supplementary material for: Heat the Clock: Entrainment and Compensation in Arabidopsis Circadian Rhythms
Source: J Circadian Rhythms. 2019 May 14;17:5. doi: 10.5334/jcr.179 (PMC6524549; doi:10.5334/jcr.179)
Supplement: Figure 1. — The larger activation energy, the higher the dependence of the reaction rate on the temperature. [file jcr-17-179-s1.pdf]

Temperature dependence

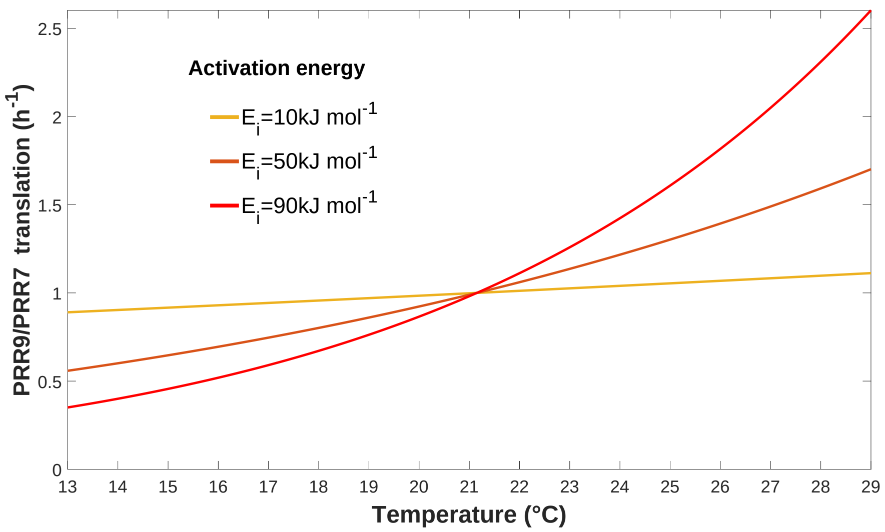

Figure 1: **The larger activation energy, the higher the dependence of the reaction rate on the temperature.** Modelled Arrhenius temperature dependence of translation rate of PRR9/PRR7 for different activation energies.
