# Supplementary material for: Heat the Clock: Entrainment and Compensation in Arabidopsis Circadian Rhythms
Source: J Circadian Rhythms. 2019 May 14;17:5. doi: 10.5334/jcr.179 (PMC6524549; doi:10.5334/jcr.179)
Supplement: Figure 2. — Incorporating the Arrhenius law allows thermal entrainment, but only within a limited temperature range. [file jcr-17-179-s2.pdf]

ELF4/LUX mRNA level

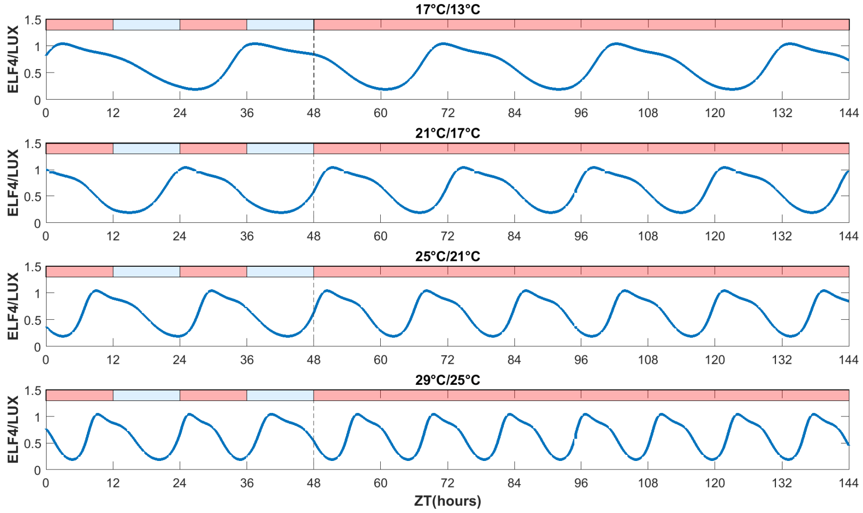

Figure 2: **Incorporating the Arrhenius law allows thermal entrainment, but only within a limited temperature range**. Periodicity of *ELF4/LUX* expression behaves similarly to the other components of the clock model under thermal conditions. A 24 h 21°C/17°C thermal cycle induces a functional clock. However, faster oscillations and ultradian rhythms are observed as temperature increases. In contrast, a 17°C/13°C thermal cycle causes notably slower oscillations.
