# Supplementary material for: Heat the Clock: Entrainment and Compensation in Arabidopsis Circadian Rhythms
Source: J Circadian Rhythms. 2019 May 14;17:5. doi: 10.5334/jcr.179 (PMC6524549; doi:10.5334/jcr.179)
Supplement: Figure 3. — Incorporating the Arrhenius law allows thermal entrainment, but only within a limited temperature range. [file jcr-17-179-s3.pdf]

PRR9/PRR7 mRNA level

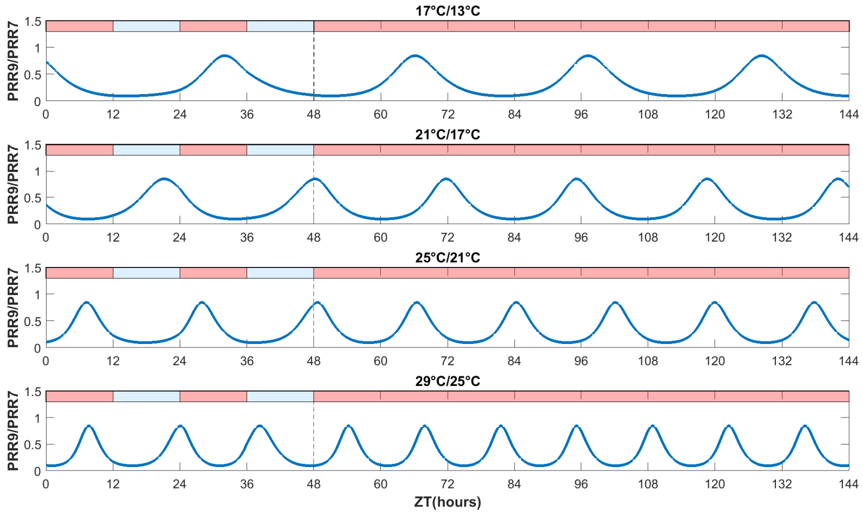

Figure 3: **Incorporating the Arrhenius law allows thermal entrainment, but only within a limited temperature range**. Periodicity of *PRR9/PRR7* expression performs comparably to the other clock components under thermal conditions. A functional clock is observed in a 24 h 21°C/17°C thermal cycle. However, faster oscillations and ultradian rhythms are obtained as temperature increases, and clearly slower oscillations are induced at 17°C/13°C thermal cycle.
