# Supplementary material for: Heat the Clock: Entrainment and Compensation in Arabidopsis Circadian Rhythms
Source: J Circadian Rhythms. 2019 May 14;17:5. doi: 10.5334/jcr.179 (PMC6524549; doi:10.5334/jcr.179)
Supplement: Figure 4. — Incorporating the Arrhenius law allows thermal entrainment, but only within a limited temperature range. [file jcr-17-179-s4.pdf]

***PRR5/TOC1* mRNA level**

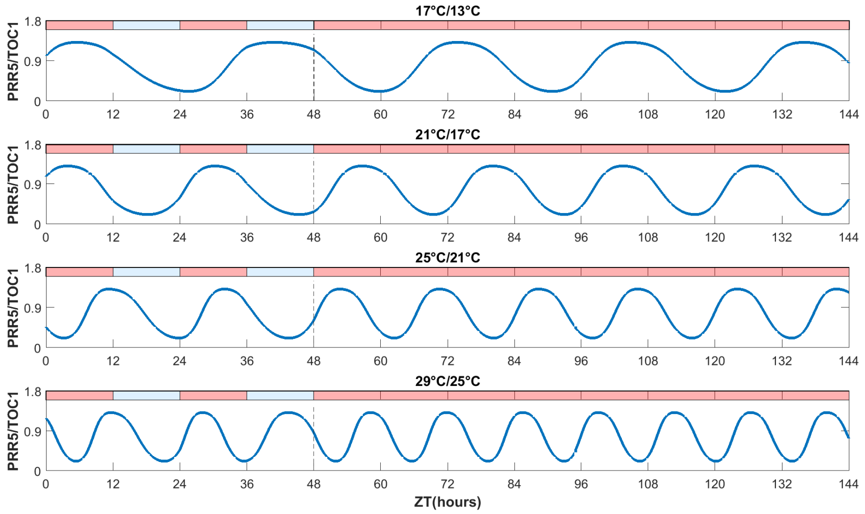

Figure 4: **Incorporating the Arrhenius law allows thermal entrainment, but only within a limited temperature range**. mRNA level periodicity of *PRR5/TOC1* agrees with the other clock component results under thermal conditions. A 24 h 21°C/17°C thermal cycle induces a functional clock. A 17°C/13°C thermal cycle causes markedly slower oscillations. However, the warmer temperature, the faster oscillations are observed.
