# Supplementary material for: Heat the Clock: Entrainment and Compensation in Arabidopsis Circadian Rhythms
Source: J Circadian Rhythms. 2019 May 14;17:5. doi: 10.5334/jcr.179 (PMC6524549; doi:10.5334/jcr.179)
Supplement: Figure 5. — Temperature stress under constant light conditions can cause a defective clock. [file jcr-17-179-s5.pdf]

Stress conditions

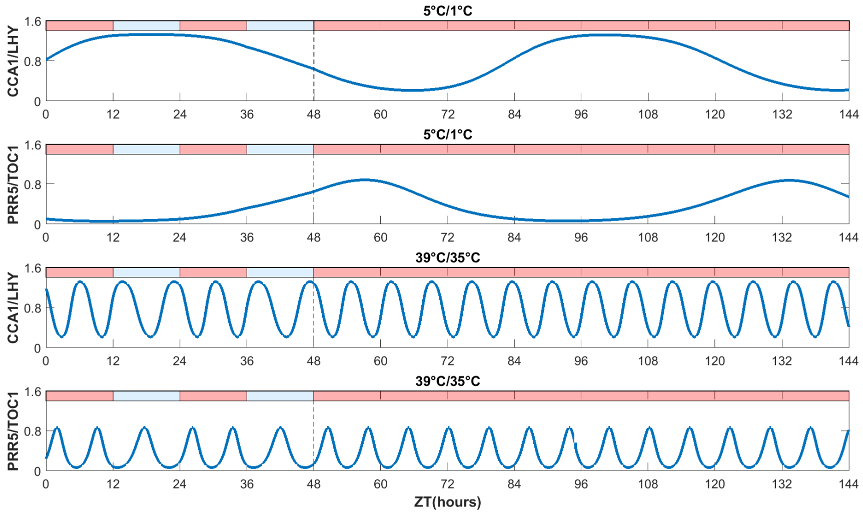

Figure 5: **Temperature stress under constant light conditions can cause a defective clock**. *CCA1/LHY* and *PRR5/TOC1* expression responses to freezing and heat stress conditions are consistent with results in Figure 2. Faster oscillations are strongly induced by heat while a freezing thermal cycle results in a heavily decreased period.
