# Supplementary material for: Heat the Clock: Entrainment and Compensation in Arabidopsis Circadian Rhythms
Source: J Circadian Rhythms. 2019 May 14;17:5. doi: 10.5334/jcr.179 (PMC6524549; doi:10.5334/jcr.179)
Supplement: Figure 6. — Thermal entrainment is observed in the model across a range of parameter values describing temperature dependence. [file jcr-17-179-s6.pdf]

Random activation energy values

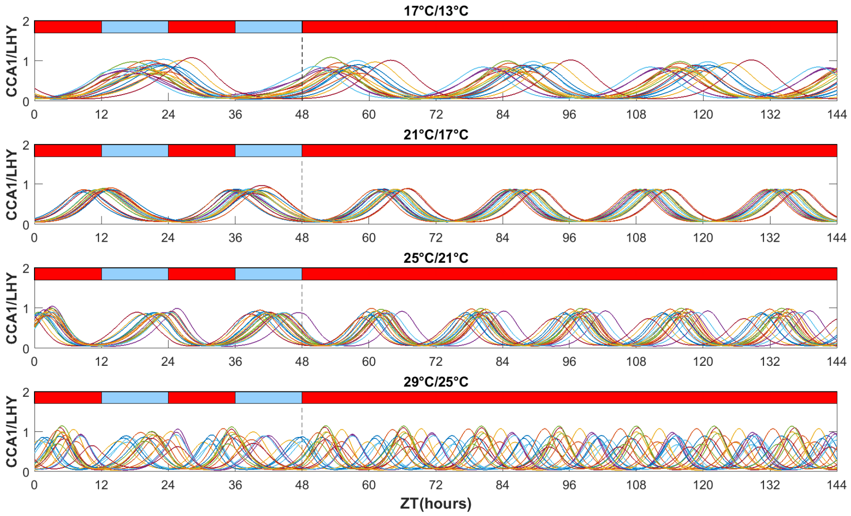

Figure 6: **Thermal entrainment is observed in the model across a range of parameter values describing temperature dependence.** Random uniform distributed activation energy values between 40 and 60 were allocated independently to each rate in the model. Results are similar to the outputs obtained when the influence of temperature was parametrized to be equal. A 24 h 21°C/17°C thermal cycle induces a functional clock. However, the warmer temperatures, the faster oscillations and ultradian rhythms are observed. In contrast, colder temperatures induce slower oscillations.
