# Supplementary material for: Heat the Clock: Entrainment and Compensation in Arabidopsis Circadian Rhythms
Source: J Circadian Rhythms. 2019 May 14;17:5. doi: 10.5334/jcr.179 (PMC6524549; doi:10.5334/jcr.179)
Supplement: Figure 7. — Incorporating temperature dependence in a more complex model [27] supports the conclusions obtained using the [11] model. [file jcr-17-179-s7.pdf]

Pokhilko et al. 2012

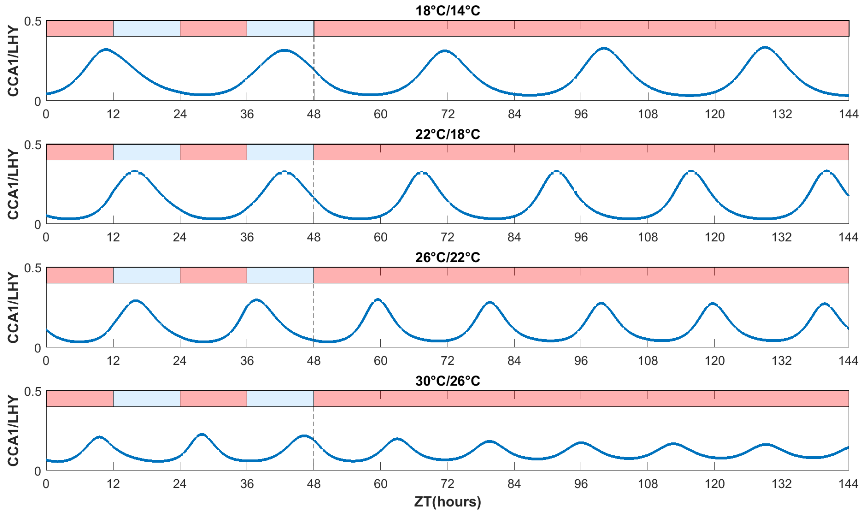

Figure 7: **Incorporating temperature dependence in a more complex model [27] supports the conclusions obtained using the [11] model.** A 24 hour 22°C/18°C thermal cycle induces a functional clock. However, colder temperatures (18°C/14°C thermal cycle) cause an increased period while warmer temperatures occasion faster oscillations and a decreased amplitude of the gene expression. To allow model comparison, temperature dependence in [27] was added to the same set of transcription, translation, and degradation parameters as those in the simpler model [11] using the same activation energies.
