# Supplementary material for: Heat the Clock: Entrainment and Compensation in Arabidopsis Circadian Rhythms
Source: J Circadian Rhythms. 2019 May 14;17:5. doi: 10.5334/jcr.179 (PMC6524549; doi:10.5334/jcr.179)
Supplement: Figure 8. — The [27] model is not temperature compensated, and this failure to compensate is driven by degradation rates. [file jcr-17-179-s8.pdf]

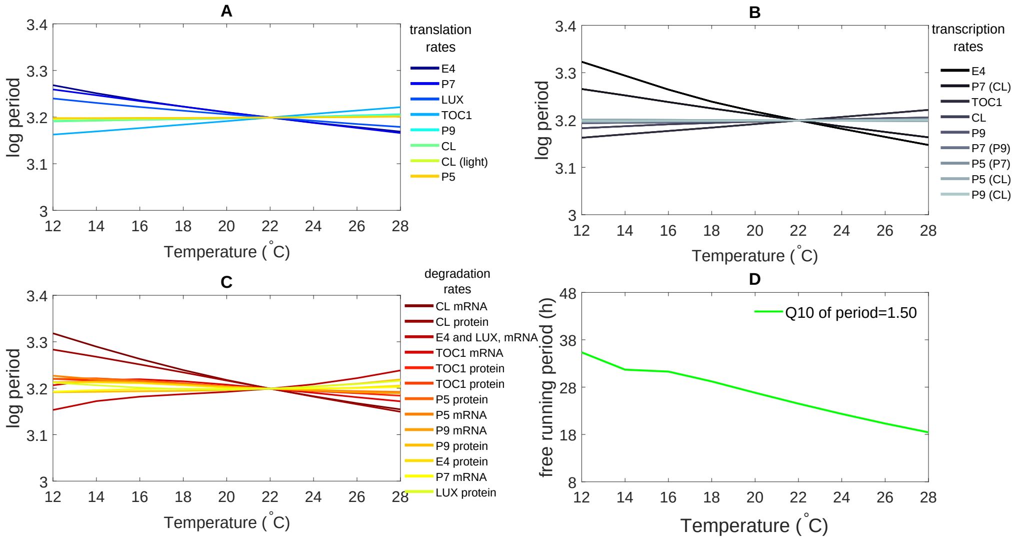

Figure 8: **The [27] model is not temperature compensated, and this failure to compensate is driven by degradation rates**. Experimental protocols of [40] and [53] were simulated. A-C show the log period across a range of temperature, where only one parameter is subject to temperature variation for each output. Results are grouped to show thermal dependence of (A) translation, (B) transcription, and (C) mRNA and protein degradation rates. Labels are in order of the size of effect and they show how the parameters' effects are induced (in brackets). The resultant free-running period when all rates vary with temperature is shown in (D). The results are qualitatively similar to those from the [11] model; thermal dependence in translation or transcription rates may either increase or decrease period. This results in a Q_{10} of period lying in the range considered for compensation. However, the effect of changing degradation rates is an overall loss of compensation.
