# Supplementary material for: Heat the Clock: Entrainment and Compensation in Arabidopsis Circadian Rhythms
Source: J Circadian Rhythms. 2019 May 14;17:5. doi: 10.5334/jcr.179 (PMC6524549; doi:10.5334/jcr.179)
Supplement: Figure 9. — The combined effect of photic and temperature forcing can induce entrainment across a wide range of light/dark durations, but only within narrow temperature limits. [file jcr-17-179-s9.pdf]

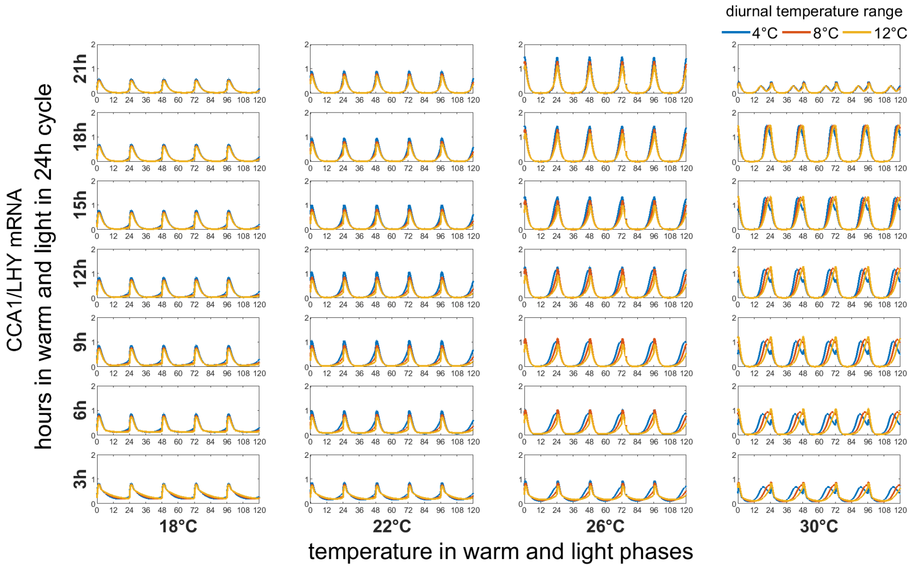

Figure 9: **The combined effect of photic and temperature forcing can induce entrainment across a wide range of light/dark durations, but only within narrow temperature limits**. Similarly to the model of [11], at 22°C the clock is correctly entrained. At lower temperatures (first column), a decreased amplitude of the gene expression is observed, and at higher temperatures (final column) there is a general increase in amplitude and non-circadian rhythms might emerge.
