# Supplementary material for: Heat the Clock: Entrainment and Compensation in Arabidopsis Circadian Rhythms
Source: J Circadian Rhythms. 2019 May 14;17:5. doi: 10.5334/jcr.179 (PMC6524549; doi:10.5334/jcr.179)
Supplement: Figure 10. — CCA1/LHY expression in prr9prr7 oscillates in response to temperature cycles with a phase shift compared to wild type. [file jcr-17-179-s10.pdf]

prr9prr7 double mutant

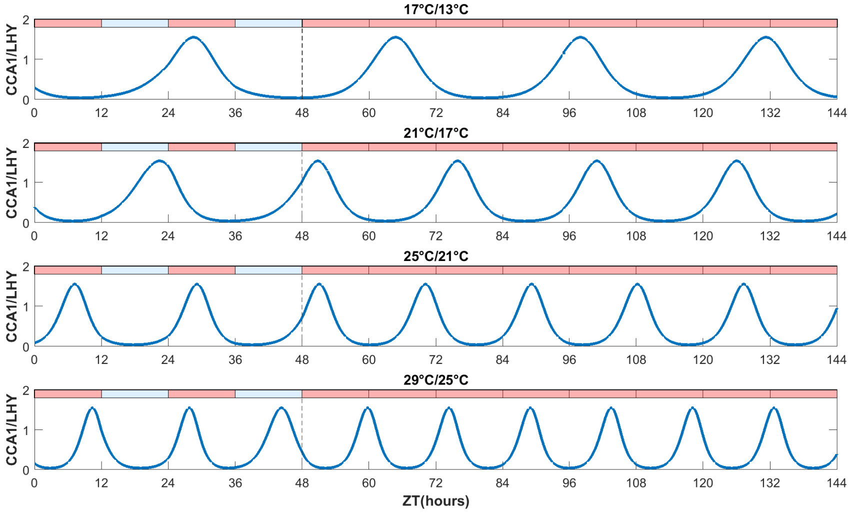

Figure 10: ***CCA1/LHY* expression in *prr9prr7* oscillates in response to temperature cycles with a phase shift compared to wild type**. Simulations were carried out similarly to Figure 2, in order to compare the outputs with the results obtained for wild type. Results are consistent with [62] observations. Oscillatory behaviour is reproduced in *prr9prr7* with expression levels peaking later than in wild type.
