# Supplementary material for: Heat the Clock: Entrainment and Compensation in Arabidopsis Circadian Rhythms
Source: J Circadian Rhythms. 2019 May 14;17:5. doi: 10.5334/jcr.179 (PMC6524549; doi:10.5334/jcr.179)
Supplement: Figure 11. — A Q10 of period of around 2 is obtained when random activation energy values between 40 kJ mol–1 and 60 kJ mol–1 are used. [file jcr-17-179-s11.pdf]

Temperature influence

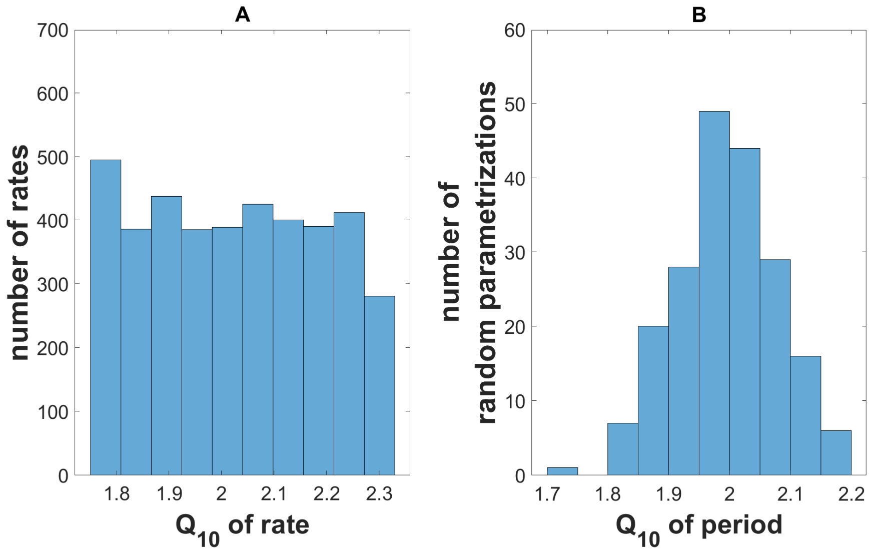

Figure 11: **A Q_{10} of period of around 2 is obtained when random activation energy values between 40 kJ mol^{-1} and 60 kJ mol^{-1} are used**. Figure **(A)** shows the distribution of the modelled inputs for 200 trajectories when random parametrizations were implemented. **(B)** displays the distribution of the modelled outputs of those trajectories.
