# Supplementary material for: Heat the Clock: Entrainment and Compensation in Arabidopsis Circadian Rhythms
Source: J Circadian Rhythms. 2019 May 14;17:5. doi: 10.5334/jcr.179 (PMC6524549; doi:10.5334/jcr.179)
Supplement: Figure 12. — CCA1/LHY expression for a diurnal temperature range of 10°C. [file jcr-17-179-s12.pdf]

Thermal cycles for a diurnal temperature range of 10°C

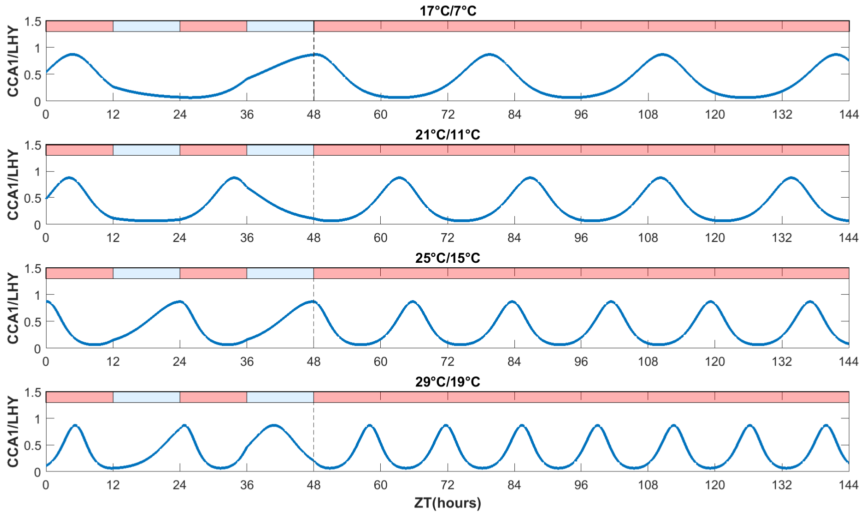

Figure 12: ***CCA1/LHY* expression for a diurnal temperature range of 10°C** . Simulations were carried out similar to Figure 2 in [11] model. Consistent with previous results, faster oscillations are observed as temperature increases. In contrast, slower oscillations are induced as temperature decreases. Unlike a diurnal temperature range of 4°C, a 24 hour thermal cycle with 25°C in warm phases induces a functional clock. A periodicity about 24 h is observed when the clock is forced. However, oscillations become faster after the clock is released, in comparison with those caused by a smaller diurnal temperature range.
