# Supplementary material for: Heat the Clock: Entrainment and Compensation in Arabidopsis Circadian Rhythms
Source: J Circadian Rhythms. 2019 May 14;17:5. doi: 10.5334/jcr.179 (PMC6524549; doi:10.5334/jcr.179)
Supplement: Figure 13. — Simulated CCA1/LHY expression qualitatively mirrors experimental data contrasting warm/light and cold/dark with cold/light and warm/dark cycles. [file jcr-17-179-s13.pdf]

Simultaneous light and temperature forcing

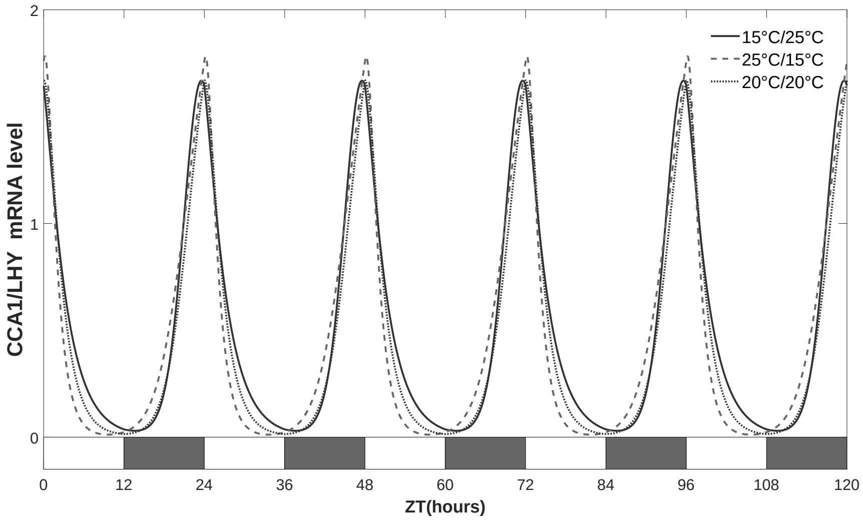

Figure 13: **Simulated *CCA1/LHY* expression qualitatively mirrors experimental data contrasting warm/light and cold/dark with cold/light and warm/dark cycles**. Simulations were carried out using a 24 h cycle combining light and temperature forcing. Light and warm, and dark and cold phases were studied in contrast with dark and warm, and light and cold conditions. These combined entraining cycles were designed to compare our results with experimental observations in [56]. Similarly to protocols used for the analysis of LHY expression in Figure 7 of [56], our results correspond to 5 days under a forced clock after 10 days of entrainment.
