# Supplementary material for: Heat the Clock: Entrainment and Compensation in Arabidopsis Circadian Rhythms
Source: J Circadian Rhythms. 2019 May 14;17:5. doi: 10.5334/jcr.179 (PMC6524549; doi:10.5334/jcr.179)
Supplement: The Model. — The model is summarised by the following system of ordinary differential equations. [file jcr-17-179-s14.pdf]

The Model

The model is summarised by the following system of ordinary differential equations.

$$\frac{d[CL]_m}{dt} = (v_1 + v_{1L} * L * [P]) * \frac{1}{1 + (\frac{[P97]_p}{K_1})^2 + (\frac{[P51]_p}{K_2})^2} - (k_{1L} * L + k_{1D} * D) * [CL]_m$$

$$\frac{d[CL]_p}{dt} = (p_1 + p_{1L} * L) * [CL]_m - d_1 * [CL]_p$$

$$\frac{d[P97]_m}{dt} = (v_{2L} * L * [P] + v_{2A} + v_{2B} * \frac{[CL]_p^2}{K_3^2 + [CL]_p^2}) * \frac{1}{1 + (\frac{[P51]_p}{K_4})^2 + (\frac{[EL]_p}{K_5})^2} - k_2 * [P97]_m$$

$$\frac{d[P97]_p}{dt} = p_2 * [P97]_m - (d_{2D} * D + d_{2L} * L) * [P97]_p$$

$$\frac{d[P51]_m}{dt} = v_3 * \frac{1}{1 + (\frac{[CL]_p}{K_6})^2 + (\frac{[P51]_p}{K_7})^2} - k_3 * [P51]_m$$

$$\frac{d[P51]_p}{dt} = p_3 * [P51]_m - (d_{3D} * D + d_{3L} * L) * [P51]_p$$

$$\frac{d[EL]_m}{dt} = L * v_4 * \frac{1}{1 + (\frac{[CL]_p}{K_8})^2 + (\frac{[P51]_p}{K_9})^2 + (\frac{[EL]_p}{K_10})^2} - k_4 * [EL]_m$$

$$\frac{d[EL]_p}{dt} = p_4 * [EL]_m - (d_{4D} * D + d_{4L} * L) * [EL]_p$$

$$\frac{d[P]}{dt} = 0.3 * (1 - [P]) * D - [P] * L$$

The parameter values are those fitted to experimental observations in [11] for a qualitative dynamics matching, summarised in Table 1, Supplementary Material.
