# Supplementary material for: Heat the Clock: Entrainment and Compensation in Arabidopsis Circadian Rhythms
Source: J Circadian Rhythms. 2019 May 14;17:5. doi: 10.5334/jcr.179 (PMC6524549; doi:10.5334/jcr.179)
Supplement: Table 1. — Parameter values. [file jcr-17-179-s15.pdf]

Parameter description	Name	Value	Units
CL transcription	v_1	4.6	nM.h^{-1}
CL light-induced transcription	v_{1L}	3.0	nM.h^{-1}
P97 transcription	v_{2A}	1.3	nM.h^{-1}
P97 CL-induced transcription	v_{2B}	1.5	nM.h^{-1}
P97 light-induced transcription	v_{2L}	5.0	nM.h^{-1}
P51 transcription	v_3	1.0	nM.h^{-1}
EL transcription	v_4	1.5	nM.h^{-1}
CL mRNA degradation (light)	k_{1L}	0.5	h^{-1}
CL mRNA degradation (dark)	k_{1D}	0.2	h^{-1}
P97 mRNA degradation	k_2	0.4	h^{-1}
P51 mRNA degradation	k_3	0.6	h^{-1}
EL mRNA degradation	k_4	0.6	h^{-1}
CL translation	p_1	0.8	h^{-1}
CL light light-induced translation	p_{1L}	0.4	h^{-1}
P97 translation	p_2	1.0	h^{-1}
P51 translation	p_3	0.6	h^{-1}
EL translation	p_4	1.0	h^{-1}
CL degradation	d_1	0.7	h^{-1}
P97 degradation (dark)	d_{2D}	0.5	h^{-1}
P97 degradation (light)	d_{2L}	0.3	h^{-1}
P51 degradation (dark)	d_{3D}	0.5	h^{-1}
P51 degradation (light)	d_{3L}	0.8	h^{-1}
EL degradation (dark)	d_{4D}	1.2	h^{-1}
EL degradation (light)	d_{4L}	0.4	h^{-1}
Inhibition: CL by P97	K_1	0.2	nM
Inhibition: CL by P51	K_2	1.2	nM
Activation: P97 by CL	K_3	0.2	nM
Inhibition: P97 by P51	K_4	0.2	nM
Inhibition: P97 by EL	K_5	0.3	nM
Inhibition: P51 by CL	K_6	0.5	nM
Inhibition: P51 by itself	K_7	2.0	nM
Inhibition: EL by CL	K_8	0.4	nM
Inhibition: EL by P51	K_9	1.9	nM
Inhibition: EL by EL	K_{10}	1.9	nM

Table 1: Parameter values.
